# Sulfur amino acid restriction, energy metabolism and obesity: a study protocol of an 8-week randomized controlled dietary intervention with whole foods and amino acid supplements

**DOI:** 10.1186/s12967-021-02824-3

**Published:** 2021-04-15

**Authors:** Emma Stolt, Thomas Olsen, Amany Elshorbagy, Viktor Kožich, Marleen van Greevenbroek, Bente Øvrebø, Magne Thoresen, Helga Refsum, Kjetil Retterstøl, Kathrine J. Vinknes

**Affiliations:** 1 Department of Nutrition, Institute of Basic Medical Sciences, University of Oslo, Sognsvannveien 9, 0372 Oslo, Norway; 2 Department of Pharmacology, University of Oxford, Oxford, UK; 3 Department of Physiology, Faculty of Medicine, University of Alexandria, Alexandria, Egypt; 4 Department of Pediatrics and Inherited Metabolic Disorders, First Faculty of Medicine, Charles University and General University Hospital, Prague, Czech Republic; 5 Department of Internal Medicine and CARIM School of Cardiovascular Diseases, Maastricht University, Maastricht, The Netherlands; 6 Department of Sports Science and Physical Education, Faculty of Health and Sport Sciences, University of Agder, Kristiansand, Norway; 7 Department of Biostatistics, Institute of Basic Medical Sciences, University of Oslo, Oslo, Norway; 8 The Lipid Clinic, Oslo University Hospital, Oslo, Norway

**Keywords:** Methionine restriction, Cysteine restriction, Sulfur amino acids, Dietary intervention, Plasma biomarkers, Translational research, Adipose tissue, Gene expression, Obesity, Metabolic health

## Abstract

**Background:**

Dietary sulfur amino acid (SAA) restriction is an established animal model for increasing lifespan and improving metabolic health. Data from human studies are limited. In the study outlined in this protocol, we will evaluate if dietary SAA restriction can reduce body weight and improve resting energy expenditure (REE) and parameters related to metabolic health.

**Method/design:**

Men and women (calculated sample size = 60), aged 18–45 years, with body mass index of 27–35 kg/m^2^ will be included in a double-blind 8-week dietary intervention study. The participants will be randomized in a 1:1 manner to a diet with either low or high SAA. Both groups will receive an equal base diet consisting of low-SAA plant-based whole foods and an amino acid supplement free of SAA. Contrasting SAA contents will be achieved using capsules with or without methionine and cysteine (SAA_high_, total diet SAA ~ 50–60 mg/kg body weight/day; SAA_low_, total diet SAA ~ 15–25 mg/kg body weight/day). The primary outcome is body weight change. Data and material collection will also include body composition (dual X-ray absorptiometry), resting energy expenditure (whole-room indirect calorimetry) and samples of blood, urine, feces and adipose tissue at baseline, at 4 weeks and at study completion. Measures will be taken to promote and monitor diet adherence. Data will be analyzed using linear mixed model regression to account for the repeated measures design and within-subject correlation.

**Discussion:**

The strength of this study is the randomized double-blind design. A limitation is the restrictive nature of the diet which may lead to poor compliance. If this study reveals a beneficial effect of the SAA_low_ diet on body composition and metabolic health, it opens up for new strategies for prevention and treatment of overweight, obesity and its associated disorders.

*Trial registration* ClinicalTrials.gov: NCT04701346, Registration date: January 8th, 2021

**Supplementary Information:**

The online version contains supplementary material available at 10.1186/s12967-021-02824-3.

## Introduction

The past decade has seen the emergence of circulating amino acids as predictors of metabolic risk, and evidence implicates a role for sulfur-containing amino acids (SAA) in obesity-related metabolic disease [1, 2]. SAA include the essential amino acid methionine, which via the transmethylation and transsulfuration pathways can be converted to cysteine, a conditionally essential amino acid [3]. Cysteine is a precursor of glutathione, taurine and H_2_S, compounds with critical metabolic properties [4], including intracellular antioxidant defense, bile acid conjugation and cardiovascular function, vasodilation, immunomodulatory and signaling functions, respectively [5].

The main sources of dietary SAA are animal-derived foods, the intakes of which are typically high in Western societies [6]. A Western diet is associated with hypertension, heart disease, obesity, diabetes, and certain cancers. In animal models, dietary SAA restriction has been demonstrated to induce several beneficial metabolic effects [7]. The first study dates back to 1993, in which methionine restriction was demonstrated to increase the lifespan of rats [8]. Subsequent studies have showed that SAA restriction results in higher energy expenditure, enhanced insulin sensitivity and lower adiposity [9, 10]. In addition, SAA restriction triggers metabolic adaptations leading to increased mitochondrial biogenesis and transcriptional remodeling of lipid and glucose metabolism with reductions in circulating and tissue lipids, hepatic steatosis, oxidative damage and inflammation [11–15]. In human observational studies, particularly circulating total cysteine linearly correlates with fat mass, independent of other SAA [16]. Collectively, dietary SAA restriction in animals and human observational data suggest that higher methionine and cysteine availability promotes fat gain [14, 17].

The substantial body of experimental data on the role of SAA as potentially causal risk factors for obesity and its metabolic sequelae has not been translated into dietary recommendations in humans, mainly because of limited data from interventions in humans. One 16-week randomized controlled trial showed that fat oxidation was increased on a methionine restricted diet [18]. Recently, we showed that a 7-day study with a SAA restricted diet decreased plasma methionine, cystathionine, urinary total cysteine, and increased serum fibroblast growth factor 21 (FGF21) and lipogenic mRNAs in adipose tissue [19]. Because of the limited human data available, effects of dietary SAA restriction should be further addressed to translate beneficial findings from animal studies to human populations with overweight and obesity. In the trial branch of the *Sulfur amino acids, energy meTAbolism and obesitY* (STAY)-project we will perform a dietary intervention in participants with overweight and obesity to (1) evaluate if 8 weeks of dietary SAA restriction reduces overweight and obesity, and improves body composition and energy expenditure, and (2) identify potential mechanisms by which lowering dietary SAA may change metabolism in humans. In this paper, we outline the study protocol of the trial according to the SPIRIT initiative: Standard protocol items: Recommendations for interventional trials [20] (see Additional file 1 for checklist).

## Objectives

The overall objective of the trial is to establish effects of SAA restriction on body weight and related parameters of metabolic health including body composition (lean mass, total and compartmental fat mass [visceral fat mass, subcutaneous fat mass]) and resting energy expenditure (REE) in humans. Further, we aim to characterize the response to SAA restriction in plasma biomarkers and gene expression patterns related to amino acid, sulfur compounds, lipid and glucose metabolism as well as markers of appetite regulation, inflammation, adipokines and markers of liver status. The overarching aim is to translate findings from previous animal experiments to humans. For a complete overview of the outcomes and their methods of measurement, see “Assessment of primary and secondary outcomes” and Table 1.

**Table 1:** Primary and secondary outcomes

Primary outcome	Measured by
• Body weight	• DXA

Secondary outcomes	Measured by
• Resting energy expenditure, respiratory quotient, substrate oxidation	• WRIC
• Changes in body composition including body fat compartments and lean mass	• DXA • Waist circumference • Hip circumference • Waist-hip-ratio
• Plasma and urine concentrations of SAA and related intermediates and compounds (sulfurome)	• LC–MS/MS
• Plasma lipoprotein profile, total fatty acid profile, and glucose, c-peptide and insulin changes	• LC–MS/MS, GC–MS/MS, ELISA, colorimetric and/or enzymatic methods
• Adipokines and satiety hormones	• ELISA
• mRNA expression of enzymes involved in SAA, lipid and energy metabolism in adipose tissue and white blood cells	• qPCR and untargeted analysis (mRNA sequencing)
• Vitamin status, including plasma folate and B12 and methylmalonic acid	• LC–MS/MS
• 24 h-urine urea nitrogen for nitrogen balance	• Modified Kjelldahl method • BUN-assay
• Changes in plasma biomarkers	• Untargeted analysis (metabolomics)
• Serum changes in FGF21	• ELISA
• Changes in markers of liver status: ALAT, ASAT, ɤ-GT, LD, ALP, CK and bilirubin	• Colorimetric and/or enzymatic methods
• Changes in gut microbiota	• Sequencing of fecal samples

*BUN* blood urea nitrogen, *DXA* dual-energy x-ray absorptiometry, *FGF21* fibroblast growth factor 2, *WRIC* whole-room indirect calorimetry, *qPCR* quantitative polymerase chain reaction, *ELISA* enzyme-linked immunosorbent assay, *MS, LC–MS/MS* liquid chromatography–mass spectrometry-tandem, *MS, GC–MS/MS* gas chromatography/mass spectrometry-tandem, *ALAT* alanine aminotransferase, *ASAT* aspartate aminotransferase, *ɤ-GT* gamma-glutamyltransferase, *LD* lactate dehydrogenase, *ALP* Alkaline phosphatase ALP, *CK* creatine kinase

## Methods and trial design

### Study setting and design

The study will be conducted at the Centre for Clinical Nutrition (CCN) at Institute of Basic Medical Sciences, University of Oslo (Oslo, Norway). This randomized controlled trial (RCT) will follow the Consolidated Standards of Reporting Trials (CONSORT) guidelines [21]. Both randomized groups will receive a full diet intervention with provision of meals and snacks for the duration of the study. The intervention starts after a 7-day run-in period, and lasts for 8 weeks. Participants will attend the CCN at baseline (week 0) and at week 4 and at the final visit (week 8) after start of intervention for clinical assessment, anthropometric measurements and for collection of biological samples. Follow-up conversations will be conducted by telephone and e-mail on a regular basis.

### Participants and eligibility

**Fig. 1:**
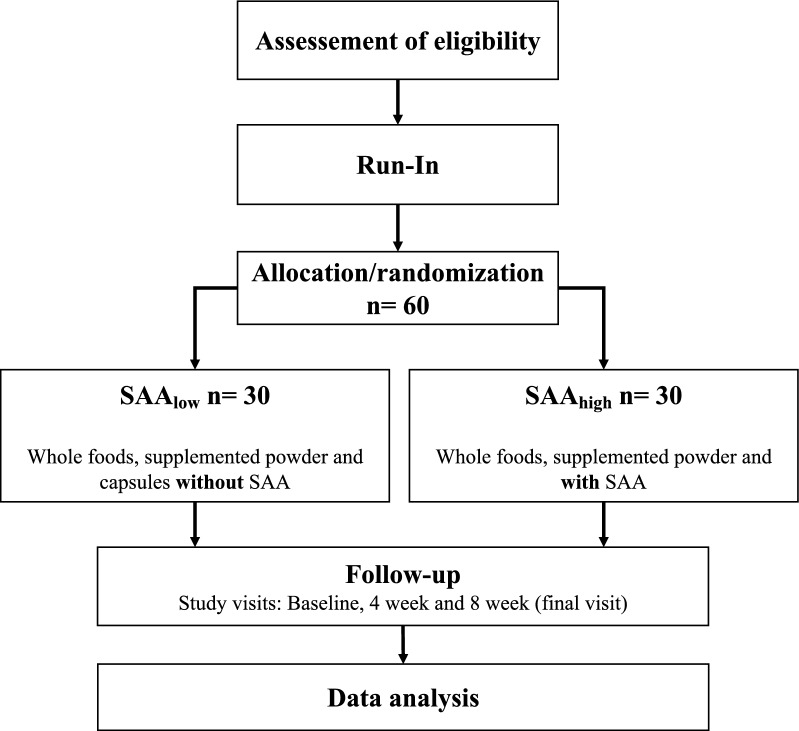
Flowchart of the study. *SAA* sulfur amino acids

Recruitment is scheduled for March 2021 through March 2022 and men and women 18–45 years of age will be included. A timeline is illustrated in Table 2, and a flow chart outlining participant flow in Fig. 1. The upper age cut-off is set to avoid heterogeneity as blood concentrations of glutathione, which is closely associated with cysteine metabolism, decrease with increasing age, possibly due to higher requirements [22]. Healthy participants with overweight and obesity (BMI 27–35 kg/m^2^) will be recruited to participate in the trial. For screening purposes and in order to ensure that potential participants with BMI between 27 and 30 are not erroneously included due to high lean mass and low body fat percentage, they will be asked to additionally report their waist circumference (WC). Traditional WC cut-offs for the definition of overweight are > 80 and > 94 cm, for women and men respectively [23]. A follow-up phone call will be made to verify all the reported information. Screening will be performed 1–3 weeks prior to baseline. Exclusion criteria include smoking, suffering from any chronic disease, established co-morbidities, veganism (≥ 1 month), pregnancy or breastfeeding the last 3 months, significant weight loss (≥ 5%) over the last 3 months and high intensity training (interval running, crossfit, heavy strength training) more than 3 session per week. An overview of the inclusion and exclusion criteria is given in Table 3.

**Table 2:**
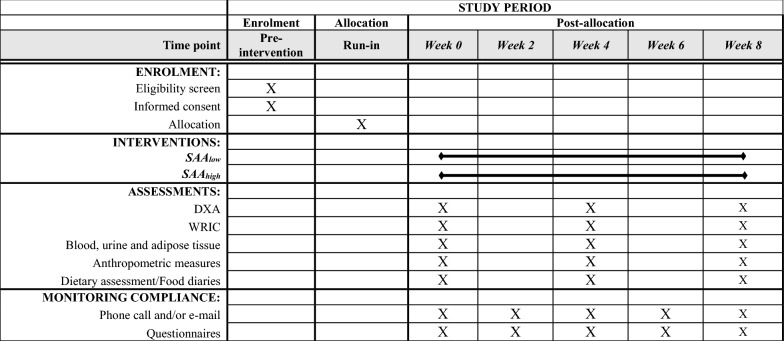
Content for the schedule of enrolment, intervention and assessments according to SPIRIT requirements

*DXA* dual X-ray absorptiometry, *SAA*_*low*_ diet low in methionine and cysteine, *SAA*_*high*_ diet high in methionine and cysteine, *WRIC* whole-room indirect calorimetry

**Table 3:** Inclusion and exclusion criteria

Inclusion	Exclusion
Age 18–45	Smoking
BMI 27–35 kg/m^2^	Chronic disease + co-morbidities
Healthy participants	Veganism (≥ 1 month)
	Pregnancy
	Breastfeeding the last 3 months
	Significant weight loss (≥ 5%) over the last 3 months
	High intensity training (interval running, Crossfit, heavy strength training) more than 3 sessions per week
Waist circumference > 80 cm for women and > 94 cm for men	

Recruitment of participants will primarily occur through social media campaigns accompanied by an informative web article about potential benefits of plant-based diets with low SAA content, and a screening questionnaire. If eligible, a follow-up phone call will be made to confirm and clarify information regarding inclusion and exclusion criteria. Eligible participants will sign an informed consent (Additional file 2, in Norwegian) where project design and measures for data collection, storage and future use are described. Data will be stored safely in an encrypted space provided by Services of Sensitive Data at the University of Oslo. A biobank for long-term storage of biological material will be created for future exploratory analyses. The study will be conducted according to the guidelines in the Declaration of Helsinki, and has been approved by the Regional Ethics Committee for Medical Research in South East Norway (Reference No: 123644) and the Norwegian Centre for Research Data (Reference No: 723047) in line with the current regulations on data protection.

### Procedures for run-in and prior to study visits

During the run-in week, participants will be asked to do the following: limit alcohol intake to comply with the Nordic Nutrition Recommendations (NNR, max. 5 g/days for women, max. 10 g/days for men); maintain their level of habitual physical activity with no more than 3 high-intensity sessions per week; and cease dietary supplementation. The participants will be encouraged to follow these general guidelines for the duration of the study. Twenty-four hours prior to the study visits, participants will be asked to avoid strenuous physical activity as well as caffeine and alcohol intake to comply with protocols for measurements of REE in the whole room indirect calorimeter (WRIC). In addition, they will be asked to avoid all food and liquid (except water) intake 12 h prior to study visits. Participants will be instructed to collect 24 h-urine, spot urine and a fecal sample prior to each visit.

### Randomization and methods against bias

Recruited participants will be randomized in a stratified block fashion to one of the two intervention arms to ensure similar numbers of participants in each group (Fig. 1). Block sizes will be random and a multiplicative of two (the number of intervention arms). Because body composition and SAA metabolism differ by sex [24], the randomization will be stratified to ensure equal number of males and females in each intervention arm. Randomization will be performed by a researcher unrelated to the study using the “blockrand” package in the R software (R Foundation for Statistical Computing, Vienna, Austria). The randomization code will be kept separate from the researchers and analysts until the end of study.

Blinding of the study participants and investigators is usually not possible in a dietary intervention. However, this trial will be double blind, since the base diet is identical for both groups and the only difference is whether they receive capsules with or without methionine and cysteine. Data analysts will be blinded to the group allocation during analysis of the primary outcome.

### Dietary interventions

#### Base diets and amino acid supplement

The base diet is identical in both intervention groups and consist of low-SAA plant-based whole foods and an amino acid powder without methionine and cysteine (XMET XCYS Maxamaid, Nutricia Norway AS, Oslo, Norway). The amino acid powder is used to achieve adequate protein intakes as the whole foods in the diet are relatively low in protein. The base diet including the powder provides approximately 15–25 mg/kg body weight of SAA per day. SAA content will be adjusted accordingly using capsules (see *Controlling the SAA content of the intervention* for details). The total energy content is 10460 kJ (2500 kcal) for men, and 9200 kJ (2200 kcal) for women. Nutrient and energy intakes are in line with the NNR [25], and calculated to not be hypocaloric. The total amount of energy/protein and SAA in the diets only differ by sex and not individual body weight. The protein intake will be ~ 0.8–1.2 g/kg body weight depending on the body weight of the participant.

A typical daily menu is given in Table 4. Methionine and cysteine are abundant in animal-derived protein and in certain fruits, grains, nuts and vegetables. Thus, the base diet is vegan-based without meat, fish, eggs, dairy products and certain plant-based foods. A list of low SAA-containing foods will be provided for the participants with the option for extra or ad libitium consumption of foods (Table 5). The foods from this list may also be used to supplement all recipes in the meal plan. Approximate daily energy content of the base diets including the powder will range from 45–60 E% from carbohydrates; 25–40 E% from fats and 10–20 E% from protein which is in line with the NNR. Regarding intake of alcohol, the participants will be advised to not exceed intake of 10 g/days for men and 5 g/days for women which is recommended by the NNR.

**Table 4:** Example of a daily menu

Meal	Ingredients/foods
Breakfast	Oat meal and/or bread with fruits
Lunch	Salad with beans, different fruits and vegetables and vegetable oil and bread
Dinner	Vegetable casserole/vegetable soup/bean salad and bread
Supper	Vegetable soup or salad with bread
Snack	Nuts/fruits

2–4 amino acid powder free of methionine and cysteine is to be consumed as drinks throughout the day

**Table 5:** List of extra foods

Foods		
Vegetables	Fruit	Spice/herbs/sweeteners
Iceberg lettuce	Pear	Dill, raw
Celery sticks	Plum	Chili red, raw
Tomato	Nectarine	Chilipowder
Onion	Strawberries	Pepper seasoning
Cabbage	Apricot	Curry powder
Mushroom	Watermelon	Basil, dried
Cucumber	Apple (2 apples/day)	Basil, raw
Eggplant		Oregano, dried
Asparagus		Apple cider vinegar
Sweet pepper, red		Lime/lime juice
Radish		Figs (as a sweetener)
Cauliflower (1 serving/day)		
Olives (10 pieces/day)		
Spinach		

Animal-derived foods are major sources of vitamin B12, and thus vitamin B12 depletion in persons following a vegan or vegetarian diet is always a concern [26]. To ensure sufficient intake of both macro and micronutrients, both groups will in addition receive a powdered drink mix without methionine and cysteine. The powder contains a balanced amount of essential and non-essential amino acids, carbohydrates, vitamins, minerals and trace elements. Flavor enhancers (Flavour Modjul® from Nutricia or an alternative enhancer selected from a pre-defined list) may be added to the drinks to mask the unpleasant taste of the mix.

#### Controlling the SAA content of the intervention

To regulate SAA content of the diets, capsules with (SAA_high_) or without (SAA_low_) methionine and cysteine will be provided in a double-blind manner. The SAA_low_ group will receive capsules with no SAA (3000 mg/days maltodextrin) (capsules: Capsuline, Florida, USA; maltodextrin: (Star Nutrition, Sweden), whereas the SAA_high_ group will receive capsules with methionine (1125 mg/days) and cystine (2500 mg/days) (Jo Mar Laboratories, Scotts Valley, CA, USA).

Total intake of SAA from base diet, powder and capsules is 50–60 mg/kg/days SAA and 15–25 mg/kg/days in the SAA_high_ and SAA_low_ group, respectively. In human and animal studies, SAA restriction has usually involved ~ 80% reduction of SAA intake compared to the control group [7]. Notably, data on the normal range of SAA intakes in humans are limited. One paper by Nimni et al*.* estimated that Western and high-protein diets contain up to 50–70 mg/kg body weight/day of SAA in total, in comparison with ~ 20–30 mg/kg body weight/day in vegans/vegetarians [6]. The SAA restricted diet in our planned intervention will meet the WHO recommendations of SAA content of a minimum of 15 mg/kg body weight/day [27].

### Assessment of primary and secondary outcomes

The primary outcome is between-group change in body weight (Table 1). Body weight is measured by the Dual-energy x-ray absorptiometry (DXA) procedure (Lunar iDXA, GE Healthcare Lunar, Buckinghamshire, United Kingdom) giving total body mass in kilograms. The secondary outcome measures include energy expenditure measured in a WRIC to calculate REE [28], body composition by DXA, body anthropometry (WC, hip circumference, height) and a range of metabolites related to SAA metabolism, other amino acids, lipid parameters, appetite hormones and gene expression profiles in adipose tissue to explore additional potential benefits of an SAA restricted diet. Methods and validation for measurements have been published for amino acid profiles [29], sulfur metabolite/aminothiol analyses including hydrogen sulfide (H_2_S) [30], total fatty acid profile [31], appetite hormones and adipokines [32, 33]. Glucose and insulin will be measured as part of the routine clinical laboratory panel for calculation of Homeostatic Model Assessment for Insulin Resistance (HOMA). This panel also includes C-peptide. Liver biomarkers including alanine aminotransferase (ALAT), aspartate aminotransferase (ASAT), lactate dehydrogenase (LD), creatine kinase (CK), alkalic phosphatases (ALP), gamma-glutamyltransferase (ɤ-GT) and the fatty liver index (based on BMI, WC, triglycerides and ɤ-GT) will be assessed to evaluate liver function and hepatic steatosis [34]. Nitrogen balance will be assessed using established methods [35, 36]. Outcome information will be collected at baseline, after 4 and at the final visit.

### Assessment of baseline diet

To obtain an estimate of the habitual diet of the participants and the magnitude of change in foods and nutrient composition due to the dietary intervention, they will complete a 279-item validated web-based FFQ at baseline [37].

### Monitoring compliance and feasibility

Compliance will be monitored during the intervention using food diaries four days including the day with the free ad libitum meal prior to each study visit as well as compliance questionnaires. The participants will be asked to note which meals or parts of them they did not consume, and if other food items not in the plan have been consumed. The participants’ subjective rating and evaluation of the diets will be collected with questionnaires including visual analogue scales at each study visit. We will measure plasma and urine SAA to objectively monitor compliance. In addition, participants will be asked to return boxes containing capsules.

### Strategies to maintain compliance

The compliance to the dietary interventions or dietary advice is a considerable source of bias. In previous pilot data, the compliance was shown to be > 80% to dietary interventions, which is likely due to the provision of all intervention foods, and the relatively short intervention period of 7 days [19]. We will continue home delivery of foods in the base diet to increase the incentive to adhere to the plan. This delivery will also include a menu with recipes for each meal and information on daily intakes.

After screening and prior to baseline, each participant will be carefully educated through nutritional counselling with study dietitians or dietitian students in their final year of training. During the intervention, the participants will be offered regular consultations with the study dietitians per telephone for encouragement and continuous evaluation of participant adherence. During these phone calls, participants will receive counseling and advice to facilitate motivation and diet adherence. They will also be allowed to ask any questions relating to the intervention, meals and recipes, and raise any concerns. In general, study personnel will be available to the participants via e-mail regarding the intervention at all times.

To limit the effects on the participants’ social life during a restrictive diet, they will be allowed one ad libitum meal per week, but this meal should precede testing days by no less than three days. The content and amount of the ad libitum meals should be noted in the food diaries. For the final week of the intervention, the participants should adhere fully to the study protocol including the meal plan.

### Sample size

Little is known about the effects of a SAA restricted diet on body weight and we have thus based power calculations on data from generic vegan interventions [38]. The online tool GLIMMPSE (https://v2.glimmpse.samplesizeshop.org) was used for power calculations [39]. With two groups and three repeated measures we will test for a significant group by time interaction in the study, indicating differences in body weight over time between the groups. Desired power is 0.8 and alpha was set to 0.05. A body weight difference of 3 kg between groups at the end of the study is considered clinically meaningful. The average body weight at baseline was set to 90 (SD 6) kg. A mean body weight difference of 1 and 3 kg between the SAA_low_ and SAA_high_ after 4 weeks and 8 weeks was set in the power calculations, respectively. Baseline body weight is generally strongly correlated with body weight at subsequent visits. We hypothesize a correlation of 0.9 between repeated measurements of body weight in each subject at each study visit and a decay rate of this correlation coefficient of 0.5 per visit. This yielded a total sample size of 46. Accounting for 25% dropout (46/1 − 0.25), the total sample size is 60 corresponding to 30 participants per group. The design file used for sample size calculations is available in the supplementary material and can be uploaded to GLIMMPSE to view results and design choices (Additional file 3).

### Statistical analysis

Our study design includes two intervention groups and three repeated measurements (baseline, 4 weeks, final visit). To control for correlated observations per subject per visit, the analysis of outcomes will be by linear mixed model regression. This model will include the treatment covariate, the visit covariate and their interaction term and a random term to control for within-subject correlation. The primary outcome variable is change in body weight. The interaction term will indicate change over time in body weight by intervention group. Depending on the pattern of missing observations and loss to follow-up, missing values will be handled with multiple imputations or model approaches (including linear mixed models which are robust to missing observations), and sensitivity analyses will be performed as recommended [40, 41].

We expect that there will be treatment heterogeneity indicating that effects may differ depending on covariate values at baseline. For example, baseline body weight is highly predictive and correlated to body weight change during an intervention. All models will thus be adjusted for the baseline values of the outcome variable. In addition, the models will be adjusted for the blocking factor used in randomization.

The analyses will be performed according to the intention-to-treat approach, but per-protocol (in which noncompliant participants are excluded) analyses will also be reported as recommended by the CONSORT guidelines [21].

### Stopping rules

Routines are implemented to identify adverse effects of the dietary interventions. Firstly, routine laboratory measures of appropriate blood biomarkers will be obtained (e.g., ASAT, ALAT, ɤ-GT, LD, creatinine) or hematological parameters including markers of B12 deficiency. Secondly, subjective measures of participant well-being are evaluated by direct questionnaires that contain questions about potential side effects, including non-specific symptoms. Individual stopping rules include allergic reactions to foods or supplement drinks, marked cardiovascular effects, fatigue or headache, or biochemical signs indicating impaired organ functions of liver, heart, kidney or blood and bone marrow.

### Strategies for data management

Data management will be handled in collaboration with the local data management team serving the Medical Faculty at the University of Oslo and handled according to the Findability–Accessibility–Interoperability and Reuse of digital assets (FAIR) principles. A data management plan has been approved by the funding organization. Data will be shared upon request and application, but will require approval from the Regional Committee for Research Ethics South-East and the Norwegian Centre for Research Data in order to comply with current privacy laws.

## Discussion

In this RCT, we will implement a dietary intervention, based on findings in animal models, to study the effects of SAA restriction on body weight, body composition and energy expenditure in humans. The diet design is similar to our previous pilot studies [19, 42] where we showed that plasma concentrations of methionine, cystathionine and urinary total cysteine decrease in SAA restricted groups along with increased serum FGF21 concentration and higher lipogenic gene expression in adipose tissue.

Presently, only one study that we are aware of has investigated the effects of methionine restriction on body composition and energy expenditure in humans [18]. This 16-weeks study with methionine restriction (but not cysteine restriction) demonstrated effects on fat oxidation, but no effects on body composition or total energy expenditure. Methionine content of the diet was controlled by using a supplement, but due to poor palatability of the supplement, 25% of the participants withdrew from the study, and poor adherence may have been a reason for the null results observed. In our pilot studies of 7 days, adherence was high, but the participants also reported poor palatability of the amino acid supplement. Thus, the restrictive nature of the diet, and poor palatability of the powder supplement make adherence a challenge in the implementation of the planned intervention. Hence, in our trial, we will take several measures to avoid drop-out. First, the strategy of the base diet has been in development over several years by personnel with nutrition background and a chef, with the aim to design a diet mainly consisting of whole foods, palatable meals, and with limited supplement intake. Second, the foods of the base diet will be delivered weekly to participant homes by a home delivery service to ensure that participants get appropriate amounts and to increase their incentive to comply. Meal plans and recipes will be sent electronically. Third, participants will be followed up on a regular basis by telephone and e-mail by study dietitians or dietitian students in their final year of training for consultation about the dietary intervention. Fourth, we will make room for an ad libitum meal per week to maintain motivation and allow social interactions with shared meals. The participants will be instructed to avoid the ad libitum meal less than three days before a study visit, as methionine metabolism after an oral methionine load may be disrupted in overweight participants [43].

### Strengths

This dietary intervention trial has several strengths, including the randomized double-blind design which is important for causal inference and a principle seldom used in human dietary intervention trials. In addition, participants are often instructed to follow a specific diet by limiting food intake or avoiding certain foods, whereas in our study, the participants will be provided with foods to ensure adherence and internal validity throughout the study. The trial will enroll healthy overweight and obese participants to ensure that effects are not influenced by the presence of disease or use of medications that may affect our results, although this selection may limit generalizability. If the diet is successful in terms of weight loss, body composition or metabolic health, it will greatly aid the translation of the findings from animal models. In addition, we can increase understanding of mechanisms underlying the beneficial effects of plant-based diets by using state-of-the-art methodology and measurement of novel potential biomarkers, including the sulfur-containing metabolites distal to cysteine metabolism [44].

### Limitations

The trial has a number of limitations. The SAA content of both the SAA_low_ and SAA_high_ diets is slightly higher than in our pilot but methionine levels are in line with Plaisance et al. [18], where effects on substrate oxidation was reported. The reason for this elevation of SAA intake is a result of slightly increasing intakes of whole foods and lowering the intake of the unpalatable supplement. The recruitment of participants through social media campaigns may result in self-selection of health-conscious participants, but this is generally a concern in all advertisement towards participants. We also note that there is a trade-off between the level of control of food intake and external validity considering that most foods will be provided to the participants at a weekly basis. For example, in everyday life, regular grocery shopping and shared meals likely expose participants to higher availability of all foods as well as impulse purchases. However, we regard this level of control necessary in order to make robust conclusions about the effects of SAA restriction on metabolic health. Future studies should evaluate the feasibility of following a SAA restricted diet in everyday life. Another limitation is that we will not be able to assess any long-term effects of SAA restriction over the course of 8 weeks. However, results from our study can hopefully inform and aid in the design of future long-term dietary interventions with dietary SAA restriction. In terms of data collection, we are unable to assess relevant complications of overweight and obesity in which SAA restriction has proved effective in animal models. For example, dietary SAA restriction may be a potential therapeutic approach to treat hepatic steatosis and improve postprandial glucose tolerance, but we do not have the resources available to assess these outcomes in a rigorous manner and therefore rely on relevant biomarkers that may may aid in the elucidation of an SAA restricted diet on the progression of these diseases (see Table 1 for details). Finally, although the diets are isoenergetic, they are not tailored in macro- and micronutrient content according to need of each participant, but we emphasize that the aim of this trial is to compare two diets with large absolute differences in SAA and not relative to energy intake.

## Conclusions

There is limited data on the effects of dietary restriction of methionine and cysteine in humans. The trial is expected to contribute high-quality scientific evidence, using state-of-the-art methodology for outcome assessment and with a focus on maintaining high participant adherence. If the results of this trial are in line with previous findings from our pilot and reports from animal studies, a plant-based diet low in methionine and cysteine may be a promising approach for weight reduction, reduced adiposity, improved metabolic health and with beneficial effects on obesity-related diseases.

## Supplementary Information

**Additional file 1. **SPIRIT checklist 2013: Recommended items to address in clinical trial protocol and related documents*.

**Additional file 2. **The informed consent letter (in Norwegian).

**Additional file 3. **Design file for power calculations.

## Data Availability

Not applicable.

## Abbreviations

ALAT: Alanine aminotransferase
ASAT: Aspartate aminotransferase
CK: Creatine kinase
CONSORT: Consolidated Standards of Reporting Trials
DXA: Dual-energy x-ray absorptiometry
ELISA: Enzyme-linked immunosorbent assay
FAIR: Findability–accessibility–interoperability and reuse of digital assets
FGF21: Fibroblast growth factor 21
ɤ-GT: Gamma-glutamyltransferase
GC–MS/MS: Gas chromatography/mass spectrometry-tandem MS
HOMA-IR: Homeostatic model of insulin resistance
LC–MS/MS: Liquid chromatography–mass spectrometry-tandem MS
LD: Lactate dehydrogenase
mRNA: Messenger ribonucleotide acid
qPCR: Quantitative polymerase chain reaction
REE: Resting energy expenditure
SAA: Sulfur amino acids
SD: Standard deviation
SPIRIT: Standard protocol items: recommendations for interventional trials
STAY: Sulfur amino acids, energy metabolism and obesity
WC: Waist circumference
WRIC: Whole room indirect calorimetry
WHR: Waist hip-ratio

## References

[1] Elshorbagy A, Jerneren F, Basta M, Basta C, Turner C, Khaled M, et al. Amino acid changes during transition to a vegan diet supplemented with fish in healthy humans. Eur J Nutr. 2016. 10.1007/s00394-016-1237-6 PMC5534203 27289540

[2] Dong Z, Sinha R, Richie JP (2018). Disease prevention and delayed aging by dietary sulfur amino acid restriction: translational implications. Ann N Y Acad Sci..

[3] Brosnan JT, Brosnan ME (2006). The sulfur-containing amino acids: an overview. J Nutr.

[4] Lu SC (2009). Regulation of glutathione synthesis. Mol Aspects Med.

[5] Sen U, Mishra PK, Tyagi N, Tyagi SC (2010). Homocysteine to hydrogen sulfide or hypertension. Cell Biochem Biophys.

[6] Nimni ME, Han B, Cordoba F (2007). Are we getting enough sulfur in our diet?. Nutr Metab (Lond).

[7] Dong Z, Sinha R, Richie JP (2018). Disease prevention and delayed aging by dietary sulfur amino acid restriction: translational implications. Ann N Y Acad Sci.

[8] Orentreich N, Matias JR, DeFelice A, Zimmerman JA (1993). Low methionine ingestion by rats extends life span. J Nutr.

[9] Stone KP, Wanders D, Orgeron M, Cortez CC, Gettys TW (2014). Mechanisms of increased in vivo insulin sensitivity by dietary methionine restriction in mice. Diabetes.

[10] Wanders D, Forney LA, Stone KP, Hasek BE, Johnson WD, Gettys TW (2018). The components of age-dependent effects of dietary methionine restriction on energy balance in rats. Obesity (Silver Spring).

[11] Malloy VL, Perrone CE, Mattocks DA, Ables GP, Caliendo NS, Orentreich DS (2013). Methionine restriction prevents the progression of hepatic steatosis in leptin-deficient obese mice. Metab Clin Exp.

[12] Hasek BE, Boudreau A, Shin J, Feng D, Hulver M, Van NT (2013). Remodeling the integration of lipid metabolism between liver and adipose tissue by dietary methionine restriction in rats. Diabetes.

[13] Ables GP, Johnson JE (2017). Pleiotropic responses to methionine restriction. Exp Gerontol.

[14] Elshorbagy AK (2014). Body composition in gene knockouts of sulfur amino acid-metabolizing enzymes. Mamm Genome.

[15] Yang Y, Wang Y, Sun J, Zhang J, Guo H, Shi Y (2019). Dietary methionine restriction reduces hepatic steatosis and oxidative stress in high-fat-fed mice by promoting H2S production. Food Funct.

[16] Elshorbagy AK, Smith AD, Kozich V, Refsum H (2012). Cysteine and obesity. Obesity (Silver Spring).

[17] Elshorbagy AK, Kozich V, Smith AD, Refsum H (2012). Cysteine and obesity: consistency of the evidence across epidemiologic, animal and cellular studies. Curr Opin Clin Nutr Metab Care.

[18] Plaisance EP, Greenway FL, Boudreau A, Hill KL, Johnson WD, Krajcik RA (2011). Dietary methionine restriction increases fat oxidation in obese adults with metabolic syndrome. J Clin Endocrinol Metab.

[19] Olsen T, Øvrebø B, Haj-Yasein N, Lee S, Svendsen K, Hjorth M (2020). Effects of dietary methionine and cysteine restriction on plasma biomarkers, serum fibroblast growth factor 21, and adipose tissue gene expression in women with overweight or obesity: a double-blind randomized controlled pilot study. J Transl Med.

[20] Chan AW, Tetzlaff JM, Altman DG, Laupacis A, Gotzsche PC, Krleza-Jeric K (2013). SPIRIT 2013 statement: defining standard protocol items for clinical trials. Ann Intern Med.

[21] Schulz KF, Altman DG, Moher D (2010). CONSORT 2010 statement: updated guidelines for reporting parallel group randomised trials. PLoS Med.

[22] Lang CA, Naryshkin S, Schneider DL, Mills BJ, Lindeman RD (1992). Low blood glutathione levels in healthy aging adults. J Lab Clin Med.

[23] Waist circumference and waist-hip ratio: report of a WHO expert consultation. 2011.

[24] El-Khairy L, Ueland PM, Nygard O, Refsum H, Vollset SE (1999). Lifestyle and cardiovascular disease risk factors as determinants of total cysteine in plasma: the Hordaland Homocysteine Study. Am J Clin Nutr.

[25] Nordic Nutrition Recommendations (2012). Integrating nutrition and physical activity.

[26] Pawlak R, Parrott SJ, Raj S, Cullum-Dugan D, Lucus D (2013). How prevalent is vitamin B(12) deficiency among vegetarians?. Nutr Rev.

[27] Consultation JWFUE. Protein and amino acid requirements in human nutrition. World Health Organ Tech Rep Ser. 2007(935):1–265, back cover. 18330140

[28] Rising R, Whyte K, Albu J, Pi-Sunyer X (2015). Evaluation of a new whole room indirect calorimeter specific for measurement of resting metabolic rate. Nutr Metab (Lond).

[29] Antoniades C, Shirodaria C, Leeson P, Baarholm OA, Van-Assche T, Cunnington C (2009). MTHFR 677 C>T Polymorphism reveals functional importance for 5-methyltetrahydrofolate, not homocysteine, in regulation of vascular redox state and endothelial function in human atherosclerosis. Circulation.

[30] Kožich V, Ditrói T, Sokolová J, Křížková M, Krijt J, Ješina P (2019). Metabolism of sulfur compounds in homocystinurias. Br J Pharmacol.

[31] Vinknes KJ, Elshorbagy AK, Nurk E, Drevon CA, Gjesdal CG, Tell GS (2013). Plasma stearoyl-CoA desaturase indices: association with lifestyle, diet, and body composition. Obesity (Silver Spring).

[32] Schipper HS, de Jager W, van Dijk ME, Meerding J, Zelissen PM, Adan RA (2010). A multiplex immunoassay for human adipokine profiling. Clin Chem.

[33] Loo BM, Marniemi J, Jula A (2011). Evaluation of multiplex immunoassays, used for determination of adiponectin, resistin, leptin, and ghrelin from human blood samples, in comparison to ELISA assays. Scand J Clin Lab Invest.

[34] Wong VWS, Adams LA, de Lédinghen V, Wong GLH, Sookoian S (2018). Noninvasive biomarkers in NAFLD and NASH—current progress and future promise. Nat Rev Gastroenterol Hepatol.

[35] Mariotti F, Tome D, Mirand PP (2008). Converting nitrogen into protein—beyond 625 and Jones' factors. Crit Rev Food Sci Nutr.

[36] Kimberly AE, Roberts MG (1905). A method for the direct determination of organic nitrogen by the Kjeldahl process. Public Health Pap Rep.

[37] Medin AC, Carlsen MH, Hambly C, Speakman JR, Strohmaier S, Andersen LF (2017). The validity of a web-based FFQ assessed by doubly labelled water and multiple 24-h recalls. Br J Nutr.

[38] Mishra S, Xu J, Agarwal U, Gonzales J, Levin S, Barnard ND (2013). A multicenter randomized controlled trial of a plant-based nutrition program to reduce body weight and cardiovascular risk in the corporate setting: the GEICO study. Eur J Clin Nutr.

[39] Kreidler SM, Muller KE, Grunwald GK, Ringham BM, Coker-Dukowitz ZT, Sakhadeo UR (2013). GLIMMPSE: online power computation for linear models with and without a baseline covariate. J Stat Softw.

[40] White IR, Horton NJ, Carpenter J, Pocock SJ (2011). Strategy for intention to treat analysis in randomised trials with missing outcome data. BMJ.

[41] Little RJ, D'Agostino R, Cohen ML, Dickersin K, Emerson SS, Farrar JT (2012). The prevention and treatment of missing data in clinical trials. N Engl J Med.

[42] Olsen T, Øvrebø B, Turner C, Bastani N, Refsum H, Vinknes K (2018). Combining dietary sulfur amino acid restriction with polyunsaturated fatty acid intake in humans: a randomized controlled pilot trial. Nutrients.

[43] Elshorbagy AK, Graham I, Refsum H (2020). Body mass index determines the response of plasma sulfur amino acids to methionine loading. Biochimie.

[44] Stipanuk MH, Ueki I (2011). Dealing with methionine/homocysteine sulfur: cysteine metabolism to taurine and inorganic sulfur. J Inherit Metab Dis.

