# Supplementary material for: Sulfur amino acid restriction, energy metabolism and obesity: a study protocol of an 8-week randomized controlled dietary intervention with whole foods and amino acid supplements
Source: J Transl Med. 2021 Apr 15;19:153. doi: 10.1186/s12967-021-02824-3 (PMC8051033; doi:10.1186/s12967-021-02824-3)
Supplement: Supplementary file 2 — Additional file 2. The informed consent letter (in Norwegian). [file 12967_2021_2824_MOESM2_ESM.pdf]

UNIVERSITETET I OSLO
DET MEDISINSKE FAKULTET

FORESPØRSEL OM DELTAKELSE I FORSKNINGSPROSJEKTET

SVOVELHOLDIGE AMINOSYRER, ENERGIMETABOLISME OG FEDME

Du mottar dette brevet i forbindelse med din henvendelse på vår studieannonsering. I samarbeid med forskere ved Avdeling for ernæringsvitenskap, Universitetet i Oslo ønsker vi å undersøke om endringer i svovelholdige aminosyrer (bestanddeler av proteiner) gjennom kosten har effekter på energiomsetningen og vektnedgang. Forskning tyder på at et redusert inntak av disse aminosyrene kan øke energiomsetningen i kroppen og dermed regulere kroppsvekt og redusere fettmassen. Vi ber deg lese gjennom informasjonen i dette brevet slik at du kan bestemme deg for om du ønsker å delta. **Det er viktig at du leser informasjonsskrivet nøye.**

HVA INNEBÆRER PROSJEKTET?

Studien er en intervensjonsstudie som er planlagt å pågå i 8 – åtte uker. Studien innebærer at du inntar måltider og næringsdrikk hver dag gjennom hele studieperioden. Studien innebærer videre oppmøte ved Avdeling for ernæringsvitenskap tre ganger i løpet av studieperioden. Dersom du blir med i studien vil du bli tilfeldig trukket ut til å delta i én av 2 grupper:

- **Gruppe 1**: Måltider og næringsdrikk rik på svovelholdige aminosyrer i 8 uker.
- **Gruppe 2**: Måltider og næringsdrikk med lavt innhold av svovelholdige aminosyrer i 8 uker.

Måltidene og næringsdrikkene i gruppe 1 og gruppe 2 er basert på et vegansk kosthold, det vil si uten kjøtt, fisk, egg, melk fra dyr. Gruppene vil ha ulikt innhold av de svovelholdige aminosyrene cystein og metionin som spesifisert over, slik at vi kan studere den spesifikke effekten av aminosyreinnhold i kosten. Du blir randomisert til en av de 2 gruppene. Randomisering betyr at du helt tilfeldig plasseres i en gruppe. Verken du eller studiepersonellet kan velge hvilken gruppe du skal plasseres i. Du har 50% sjanse til å havne i en av gruppene. Etter randomisering og underveis i studien vil derimot du og studiepersonellet få vite hvilken gruppe du tilhører.

Studien innebærer **tre** besøk på Senter for klinisk ernæring ved Avdeling for ernæringsvitenskap, Universitetet i Oslo.

I prosjektet vil vi innhente og registrere opplysninger om deg. Det vil bli tatt en del prøver og undersøkelser av deg før, under og etter studieperioden.

Prosedyrene i studien inkluderer følgende:

- Fettbiopsi ved bruk av nål (størrelse: 2,1x80 mm) gjennomføres 3 ganger. Biopsien tas fra mageregionen, på hver side. Det vil bli gitt lokalbedøvelse som virker hurtig og som gjør at du ikke vil kjenne noe. Å ta biopsi vil kunne gi informasjon om endringer genuttrykk i fettcellene som vil gi oss informasjon om kostens effekt på energiomsetningen i kroppen.
- Blodprøver. Analysene som skal utføres i blodprøvene inkluderer måling av konsentrasjoner av metionin, cystein og relaterte aminosyrer, og ulike markører knyttet til kostens effekt på energiomsetningen i kroppen. Vi skal også analysere genvarianter og uttrykk som kan være med å forklare ulik respons på kosten. I de samme blodprøvene kan det senere bli aktuelt å utføre flere analyser som har med effekten og omsetningen av metionin og cystein i kroppen.

- Urinprøver. Analysene skal brukes til å måle konsentrasjoner av metionin, cystein og relaterte aminosyrer.
- Målinger av vekt, høyde, kroppssammensetning, blodtrykk og puls
- Utfylling av spørreskjemaer som omhandler dine kostholds- og livsstilsvaner, helsetilstand og sykehistorie, og appetitt, sult og metthetsfølelse.
- 45 minutter i et lukket rom for å måle kroppens energiomsetning i hvile
- **Prøvetaking og målinger innebærer tre besøk på Senter for klinisk ernæring ved Avdeling for ernæringsvitenskap, Universitetet i Oslo. Disse vil finne sted på følgende tidspunkt: visitt 1 (første dag), visitt 2 (4 uker) og visitt 3 (8 uker). Alle visittene vil vare i 3-4 timer.**

I løpet av studien forventer vi at du overholder prosedyrene i studien og avtalte besøk, og at du følger muntlige og skriftlige instrukser fra prosjektansvarlig.

MULIGE FORDELER OG ULEMPER

Studien kan gi nyttig informasjon som kan bidra til ny kunnskap om effekten av aminosyrer på energiomsetning og vektregulering. Denne kunnskapen kan bidra til utvikling av nye behandlinger for fedme og fedmerelaterte sykdommer. Du vil selv få tilgang til alle dine resultater og vil dermed få en grundig helsesjekk ved tre måletidspunkter, vite din kroppsvekt og kroppssammensetning i tillegg til hvileforbrenning. Du vil også kunne forvente vektnedgang uavhengig av hvilken gruppe du trekkes ut til. Mat vil leveres til deg på ukentlig basis.

Den største ulempen ved å delta er at du må dra til Universitetet i Oslo for å hente næringsdrikk og bli undersøkt på undersøkelsesdagene. Dessuten kan du oppleve bivirkninger som følge av inntaket av næringsdrikken/måltidene, fettbiopsi eller blodprøvetakingen. Mulige bivirkninger av diettene kan være hodepine, svimmelhet, tretthet og mage-tarmplager. Mulige bivirkninger ved blodprøvetaking kan være smerte, blødning eller blåmerker, eller svimmelhet. Mulige bivirkninger ved fettbiopsi kan være smerte, blødning, infeksjoner, blåmerker, svimmelhet. Vennligst informer en av forskerne/prosjektmedarbeiderne om ubehag som måtte oppstå.

Studien følger plan og prosedyrer så nøyaktig som mulig, men situasjonen kan endre seg underveis. Vi informerer deg så snart det er mulig om det fremkommer ny informasjon om prosedyrene som anvendes i denne studien som kan påvirke din villighet til å delta i studien. Du avgjør deretter om du vil avbryte deltagelsen i studien eller om du vil fortsette. Hvis din sikkerhet eller helse er i fare, vil vi avslutte undersøkelsene øyeblikkelig.

FRIVILLIG DELTAKELSE OG MULIGHET FOR Å TREKKE SITT SAMTYKKE

Det er frivillig å delta i prosjektet. Dersom du ønsker å delta, undertegner du samtykkeerklæringen på siste side. Du kan når som helst og uten å oppgi noen grunn trekke ditt samtykke. Dersom du trekker deg fra prosjektet, kan du kreve å få slettet innsamlede prøver og opplysninger, med mindre opplysningene allerede er inngått i analyser eller brukt i vitenskapelige publikasjoner. Dersom du senere ønsker å trekke deg eller har spørsmål til prosjektet, kan du kontakte:

Prosjektleder:
Kathrine Vinknes
kathrine.vinknes@medisin.uio.no

Prosjektmedarbeider:
Thomas Olsen
thomas.olsen@medisin.uio.no

HVA SKJER MED OPPLYSNINGENE OM DEG?

Opplysningene som registreres om deg skal kun brukes slik som beskrevet i hensikten med prosjektet. Du har rett til innsyn i hvilke opplysninger som er registrert om deg og rett til å få korrigert eventuelle feil i de opplysningene som er registrert. Du har også rett til å få innsyn i sikkerhetstiltakene ved behandling av opplysningene.

Alle opplysningene vil bli behandlet uten navn og fødselsnummer eller andre direkte gjenkjennende opplysninger. En kode knytter deg til dine opplysninger gjennom en navneliste. Det er kun prosjektleder Kathrine Vinknes og Thomas Olsen som har tilgang til denne listen.

Opplysningene om deg vil bli anonymisert eller slettet fem år etter prosjektslutt.

DELING AV DATA OG OVERFØRINGER TIL UTLANDET

Ved å delta i prosjektet, samtykker du også til at opplysninger (ikke koblet til dine persondata) kan overføres til utlandet som ledd i forskningssamarbeid og publisering, inkludert til samarbeidende laboratorier (Tsjekkia), som vil utføre spesialiserte analyser av blod og urin. Prosjektleder vil sikre at dine opplysninger blir ivaretatt på en trygg måte og i henhold til europeisk personvernlovgivning.

Koden som knytter deg til dine personidentifiserbare opplysninger vil ikke bli utlevert.

En beskrivelse av denne studien vil være tilgjengelig på https://www.clinicaltrials.gov/ct2/show/NCT04701346, i henhold til amerikansk lovgivning. Denne nettsiden inneholder ikke informasjon som kan identifisere deg, men en beskrivelse av prosjektet og etter hvert også sammendrag av resultatene. Du kan gå inn på denne nettsiden når som helst.

HVA SKJER MED PRØVER SOM BLIR TATT AV DEG?

Prøvene som tas av deg (blod, urin og fettvev) skal oppbevares i en forskningsbiobank tilknyttet prosjektet, ved Avdeling for Ernæringsvitenskap, Universitetet i Oslo. Professor Kjetil Retterstøl, Institutt for medisinske basalfag, Det Medisinske Fakultet, Universitetet i Oslo er ansvarshavende for forskningsbiobanken.

Biobanken opphører ved prosjektslutt. Det vil bli søkt om godkjenning dersom det blir behov for å fortsette lagring av humant biologisk materiale til forskningsformål utover prosjektslutt.

Materiale som eventuelt sendes til utlandet for analysering, vil returneres eller destrueres ved prosjektslutt.

FORSIKRING

Du er forsikret i henhold til reglene om "Norsk Pasientskadeerstatning".

ØKONOMI

Sponsor (ansvarlig for studien) er Universitetet i Oslo. Studien og biobanken er finansiert gjennom forskningsmidler fra Norges Forskningsråd og Joint Programming Initiative – A Healthy Diet for a Healthy Life. Deltakerne vil få utdelt enkelte matvarer og produkter som ikke er bestemt av sponsor. Ingen enkeltprodukter vil kunne markedsføres direkte basert på resultater fra studien.

GODKJENNING

Regional komité for medisinsk og helsefaglig forskningsetikk har vurdert prosjektet, og har gitt forhåndsgodkjenning (REK-nummer: 126634).

Etter ny personopplysningslov har behandlingsansvarlig Universitetet i Oslo og prosjektleder Kjetil Retterstøl et selvstendig ansvar for å sikre at behandlingen av dine opplysninger har et lovlig grunnlag. Dette prosjektet har rettslig grunnlag i EUs personvernforordning artikkel 6 nr. 1a og artikkel 9 nr. 2a og ditt samtykke.

Du har rett til å klage på behandlingen av dine opplysninger til Datatilsynet.

KONTAKTOPPLYSNINGER

Dersom du har spørsmål til prosjektet kan du ta kontakt med:
Prosjektleder:
Kathrine Vinknes
kathrine.vinknes@medisin.uio.no

Prosjektmedarbeider:
Thomas Olsen
thomas.olsen@medisin.uio.no

Personvernombudet ved Universitetet i Oslo er Roger Markgraf-Bye. Personvernombudet kan nås via e-post: personvernombud@uio.no

JEG SAMTYKKER TIL Å DELTA I PROSJEKTET OG TIL AT MINE PERSONOPPLYSNINGER OG MITT BIOLOGISKE MATERIALE BRUKES SLIK DET ER BESKREVET

Sted og dato

Deltakers signatur

Deltakers navn med trykte bokstaver
